# Is Meropenem as a Monotherapy Truly Incompetent for Meropenem-Nonsusceptible Bacterial Strains? A Pharmacokinetic/Pharmacodynamic Modeling With Monte Carlo Simulation

**DOI:** 10.3389/fmicb.2019.02777

**Published:** 2019-11-29

**Authors:** Xiangqing Song, Yi Wu, Lizhi Cao, Dunwu Yao, Minghui Long

**Affiliations:** Department of Pharmacy, Hunan Cancer Hospital, The Affiliated Cancer Hospital of Xiangya School of Medicine, Central South University, Changsha, China

**Keywords:** meropenem, monotherapy, meropenem-nonsusceptible bacteria, meropenem-resistant bacteria, pharmacokinetic/pharmacodynamics, Monte Carlo simulation

## Abstract

Infections due to meropenem-nonsusceptible bacterial strains (MNBSs) with meropenem minimum inhibitory concentrations (MICs) ≥ 16 mg/L have become an urgent problem. Currently, the optimal treatment strategy for these cases remains uncertain due to some limitations of currently available mono- and combination therapy regimens. Meropenem monotherapy using a high dose of 2 g every 8 h (q 8 h) and a 3-h traditional simple prolonged-infusion (TSPI) has proven to be helpful for the treatment of infections due to MNBSs with MICs of 4–8 mg/L but is limited for cases with higher MICs of ≥16 mg/L. This study demonstrated that optimized two-step-administration therapy (OTAT, i.e., a new administration model of i.v. bolus plus prolonged infusion) for meropenem, even in monotherapy, can resolve this problem and was thus an important approach of suppressing such highly resistant bacterial isolates. Herein, a pharmacokinetic (PK)/pharmacodynamic (PD) modeling with Monte Carlo simulation was performed to calculate the probabilities of target attainment (PTAs) and the cumulative fractions of response (CFRs) provided by dosage regimens and 39 OTAT regimens in five dosing models targeting eight highly resistant bacterial species with meropenem MICs ≥ 16 mg/L, including *Acinetobacter baumannii, Acinetobacter* spp., *Enterococcus faecalis, Enterococcus faecium, Pseudomonas aeruginosa, Staphylococcus epidermidis, Staphylococcus haemolyticus*, and *Stenotrophomonas maltophilia*, were designed and evaluated. The data indicated that meropenem monotherapy administered at a high dose of 2 g q 8 h and as an OTAT achieved a PTA of ≥90% for isolates with an MIC of up to 128 mg/L and a CFR of ≥90% for all of the targeted pathogen populations when 50% *f* T > MIC (50% of the dosing interval during which free drug concentrations remain above the MIC) is chosen as the PD target, with *Enterococcus faecalis* being the sole exception. Even though 50% *f* T > 5 × MIC is chosen as the PD target, the aforementioned dosage regimen still reached a PTA of ≥90% for isolates with an MIC of up to 32 mg/L and a CFR of ≥90% for *Acinetobacter* spp., *Pseudomonas aeruginosa*, and *Klebsiella pneumoniae* populations. In conclusion, meropenem monotherapy displays potential competency for infections due to such highly resistant bacterial isolates provided that it is administered as a reasonable OTAT but not as the currently widely recommended TSPI.

## Introduction

The increasing emergence of meropenem-nonsusceptible bacterial strains (MNBSs), such as *Enterobacteriaceae, Pseudomonas aeruginosa*, and *Acinetobacter* spp., which are defined as any isolate displaying minimum inhibitory concentration (MIC) of 2, 4, and 4 mg/L with meropenem, imipenem, and/or doripenem, respectively [Clinical and Laboratory Standards Institute (CLSI), 2019], has become a serious global health concern (Nordmann, 2014; Iovleva and Doi, 2017; Bulens et al., 2018; Moghnieh et al., 2018; Huang et al., 2019). The resulting infections, which have increasingly been identified not only in hospitals (Snitkin et al., 2012; Onori et al., 2015) but also in the community (Kelly et al., 2017; Salomão et al., 2017), result in excessive morbidity, mortality, and costs (Lemos et al., 2014; Bartsch et al., 2017) and severely limit treatment options.

Some traditional agents, such as polymyxins, tigecycline, fosfomycin, and aminoglycosides, etc. and currently relatively novel ones, such as ceftazidime-avibactam, meropenem-vaborbactam, and imipenem/cilastatin-relebactam, etc. in monotherapy exhibit good potency for MNBSs; however, these agents are unfortunately limited by either significant shortcomings for the traditional agents (e.g., nephrotoxicity for polymyxins and aminoglycosides, increased mortality for tigecycline, and insufficient blood concentration due to the oral dosage form and dosage for fosfomycin) or geographical availability restrictions or unlisting for the novel ones (Satlin and Walsh, 2017; Karaiskos et al., 2019). Likewise, meropenem-containing combination therapy (MCCT) with synergism, which is currently being widely studied and recommended for MNBSs, mostly for meropenem-nonsusceptible *Klebsiella pneumoniae*, is also limited for the following reasons: (i) it has not been satisfactorily investigated in large-scale randomized clinical trials, despite the existence of sporadic controlled trials and *in vitro* studies on this form of therapy (Liu et al., 2016; Oliva et al., 2017; Satlin and Walsh, 2017; Paul et al., 2018), and (ii) the extrapolation of MCCT based on meropenem-nonsusceptible *K. pneumoniae* to other MNBSs with different resistance mechanisms lacks further investigation and verification; and importantly, (iii) its efficacy in certain situations remains controversial relative to monotherapy since some of them do not truly improve the clinical outcomes (Del Bono et al., 2017; Paul et al., 2018). Consequently, the choice of MCCT or monotherapy for the treatment of infections due to different MNBSs remains a matter of debate, and the optimal treatment for MNBSs remains uncertain.

Meropenem, at recommended optimal dosing conditions, is often included in treatment regimens for infections with a MNBS. Indeed, both theoretical and clinical studies have shown that an optimal regimen using a high dose of 2 g every 8 h (q 8 h) and a 3-h prolonged infusion for meropenem as monotherapy improves its efficacy against MNBSs with MICs of 4–8 mg/L (Jaruratanasirikul et al., 2015; Tumbarello et al., 2015). However, the majority of these isolates often have meropenem MICs ≥16 mg/L (Tumbarello et al., 2015; Gomez-Simmonds et al., 2016; Cojutti et al., 2018), limiting the utility of this approach. However, to the best of our knowledge, this outcome occurs because traditional simple prolonged-infusion (TSPI) leads to both a decrease in the peak concentration and a delay in the peak time, resulting in an apparent incompetency for meropenem against the isolates with MICs ≥16 mg/L. For this reason, reoptimization of the method for administering meropenem in monotherapy may continue to show promise regarding the successful management of these problematic isolates, prompting us to design a theoretically optimized two-step-administration therapy (OTAT, see OTAT design in the part of Materials and Methods) for these problematic isolates. It is believed that this reoptimization will not only be crucial for maximizing microbiological outcomes but also be particularly important when better treatment options for these pathogens strains are absent.

Given that few attempts have been made to determine the optimal treatment for meropenem as a monotherapy against the increasing number of MNBSs with MICs ≥ 16 mg/L, this study focused on only these strains. An attempt was made to design OTAT regimens for meropenem to determine whether its use as a monotherapy can achieve an acceptable pharmacokinetic (PK)/pharmacodynamic (PD) exposure to illustrate whether meropenem monotherapy is incompetent for use with these strains, with the intent of defining optimal dosage regimens for meropenem against such strains if possible.

## Materials and Methods

### Study Design

Meropenem-specific serum PK parameters were obtained from published literature and microbiological susceptibility data for the targeted pathogens were obtained from the European Committee on Antimicrobial Susceptibility Testing (EUCAST); together with dosing parameters, such as the dose and infusion time, these data were incorporated into a PK/PD model. Monte Carlo simulation was used to calculate the probabilities of target attainment (PTAs) at different MICs and the cumulative fractions of response (CFRs) for the targeted bacteria population with a pooled MIC distribution provided by each dosage regimen against 8 targeted bacterial species with doubling MICs between 16 and 512 mg/L from the EUCAST database for a given PK/PD target. A PTA or CFR of ≥90% and the causal dosage regimens were considered optimal.

### OTAT Design

**Figure 1:**
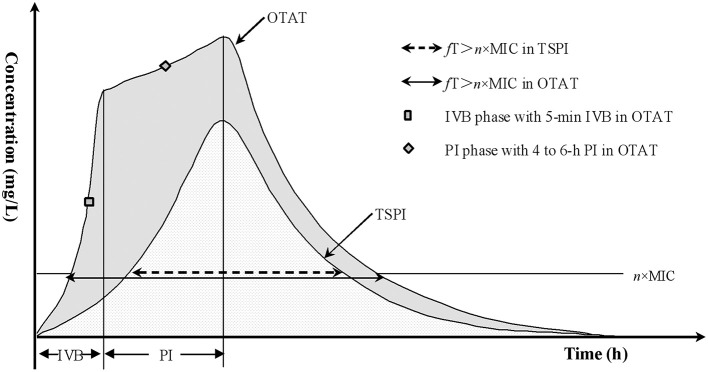
Concentration-time profiles via TSPI and OTAT. IVB, i.v. bolus; PI, prolonged infusion; OTAT, optimized two-step-administration therapy; TSPI, traditional simple prolonged-infusion; MIC, minimum inhibitory concentration; *f*, fraction of unbound drug; *f*T > *n* × MIC, the time that the unbound (free) drug concentrations remain above the MIC by *n*-fold.

Considering that the i.v. bolus (IVB) administration mode can rapidly and maximally achieve the loading drug concentration for a given agent and that prolonged infusion can continuously maintain the efficacious drug exposure, using these two techniques in combination is speculated to be optimal and competent for coverage of highly resistant bacterial isolates for a given pathogen. Therefore, OTAT represents such an administration mode in this study in which a loading dose of the total amount of the tested drug is first administered via a rapid IVB, immediately followed by the remainder of the experiment via prolonged infusion after the first dose, as illustrated in Figure 1.

### Meropenem Dosage Regimens

Meropenem dosage regimens, including the injection method, were chosen based on the licensed and studied regimens used for infected adults with normal renal function, including 0.5 g every 8 h (q 8 h), 1 g q 8 h, and 2 g q 8 h. In addition, 0.75 g every 6 h (q 6 h) and 1.5 g q 6 h were also investigated as the modified dosage regimens of 1 g q 8 h and 2 g q 8 h, respectively. Since meropenem in solutions is stable for only ~6 h at room temperature (Kuti et al., 2004), the remainder of the experiment can only be administered by prolonged infusion up to 6 h, and 4- to 6-h prolonged infusion for the remainder were thus simulated. In this study, the following 39 dosage regimens presented in Table 1 were investigated.

**Table 1:** Simulated dosage regimens for meropenem.

**Dosing model**	**Simulated dosage regimens**	
	**TSPI**	**OTAT**
0.5 g q 8 h	0.5 g (4 h)	0.25 g (5-min IVB) + 0.25 g (4 h)
	0.5 g (5 h)	0.25 g (5-min IVB) + 0.25 g (5 h)
	0.5 g (6 h)	0.25 g (5-min IVB) + 0.25 g (6 h)
1 g q 8 h	1 g (4 h)	0.5 g (5-min IVB) + 0.5 g (4 h)
	1 g (5 h)	0.5 g (5-min IVB) + 0.5 g (5 h)
	1 g (6 h)	0.5 g (5-min IVB) + 0.5 g (6 h)
2 g q 8 h	2 g (4 h)	0.5 g (5-min IVB) + 1.5 g (4 h), 1 g (5-min IVB) + 1 g (4 h), 1.5 g (5-min IVB) + 0.5 g (4 h)
	2 g (5 h)	0.5 g (5-min IVB) + 1.5 g (5 h), 1 g (5-min IVB) + 1 g (5 h), 1.5 g (5-min IVB) + 0.5 g (5 h)
	2 g (6 h)	0.5 g (5-min IVB) + 1.5 g (6 h), 1 g (5-min IVB) + 1 g (6 h), 1.5 g (5-min IVB) + 0.5 g (6 h)
0.75 g q 6 h	0.75 g (4 h)	0.5 g (5-min IVB) + 0.25 g (4 h)
	0.75 g (5 h)	0.5 g (5-min IVB) + 0.25 g (5 h)
	0.75 g (6 h)	0.5 g (5-min IVB) + 0.25 g (6 h)
1.5 g q 6 h	1.5 g (4 h)	0.5 g (5-min IVB) + 1 g (4 h), 1 g (5-min IVB) + 0.5 g (4 h)
	1.5 g (5 h)	0.5 g (5-min IVB) + 1 g (5 h), 1 g (5-min IVB) + 0.5 g (5 h)
	1.5 g (6 h)	0.5 g (5-min IVB) + 1 g (6 h), 1 g (5-min IVB) + 0.5 g (6 h)

### Meropenem Tissue PK Profiles and PD Model Associated With Clinical Response

Meropenem tissue penetration to the infection site is critical for obtaining a good clinical outcome in patients with different infection sites. Previous studies indicated that the mean meropenem tissue penetration (i.e., the mean tissue/concomitant serum concentration) after injection ranged from ~0.2 to 1 for different tissues as follows: ~0.5 for liver, lung, skin, uterus, ovaries, rectum, prostate, thyroid, trachea, and lymph nodes with the exception of a very small concentration in the brain and cerebrospinal fluid (Harrison et al., 1989), 0.95 for peritoneum (Hextall et al., 1991), 0.2 for bronchial secretions (Bergogne-Bérézin et al., 1994), 1.1 for skin exudate (Wise et al., 1990), and 0.85-0.87 for blister fluid (Mouton and Michel, 1991). However, Byl et al. (1999) reported it to be 0.17–0.43 for lung, 0.20–0.55 for bronchial mucosa, and 0.18–0.26 for pleural tissues. Regarding meropenem tissue penetration, especially for lung, a recent study conducted by Lodise et al. (2011) reported that the ratio of the area under the concentration-time curve (AUC) in epithelial lining fluid (ELF) to the AUC in plasma (AUC_ELF_/AUC_plasma_ ratio) for meropenem varied substantially between patients with ventilator-associated pneumonia, with the 10th and 90th percentile ELF exposures that were 3.7–178% of the plasma AUC values and with the mean and median AUC exposures that were 81.6 and 25.42% of the plasma values. Overall, these observations imply that logically, mean 1–2 times meropenem plasma concentrations or exposures would achieve the desired tissue concentration or exposure for most tissues if they increase proportionally, with the exception of ~5 times for lung, bronchial and pleural tissues. Theoretically, it would be more accurate to predict and establish a drug regimen based on the relationship between the tissue drug concentration and tissue exposure targets. However, to the best of our knowledge, penetration of meropenem in infected tissues to achieve the exposure targets has not been studied often, and drug concentrations in extracellular compartments are difficult to determine; thus, correlations between the PK/PD index in the tissue and antimicrobial effects are less well-understood (Nightingale and Mur, 2007). Therefore, serum PK parameters based on the plasma drug concentrations are most commonly used as surrogates for establishing and estimating the PK/PD indices in some studies (Kuti et al., 2003; Ikawa et al., 2011), and so it is with the present study.

Regarding the correlations between the PK/PD index and clinical response to meropenem therapy, Roberts et al. (2014) reported that 50 and 100% of the ratios of *f* T > MIC to a dosing interval are independent factors that influence the clinical outcome of patients receiving meropenem or other β-lactams and that a higher PK/PD index is associated with a higher likelihood of a positive clinical outcome. Likewise, Zhou et al. (2011) also found that *f* T > MIC is an independent influencing factor for predicting clinical success and that the cutoff value using *f* T > MIC based on serum concentrations in elderly patients with lower respiratory tract infections (LRTIs) is 76%. However, Li et al. (2007) studied the indices of clinical PD for LRTIs through meropenem serum concentrations in 101 patients and considered that the minimum concentration of drug in serum *f* (C_min_)/MIC > 5 rather than %*f* T > MIC is the only significant predictor of clinical response since 100% of *f* T > MIC is achieved in the majority of LRTI patients. However, there is no consensus regarding which strategy (%*f* T > MIC vs. *f* (C_min_)/MIC > 5) is better. In the present study, %*f* T > MIC is therefore used as the PD target associated with the clinical response of meropenem therapy.

Generally, 40–50% of *f* T > MIC for meropenem based on the serum concentration is usually used for predicting clinical and microbiological outcomes and for optimizing dosage regimens in most current meropenem PK/PD studies (Burgess et al., 2007; Watanabe et al., 2007; Ikawa et al., 2011; Kondo et al., 2014). However, given the profiles of meropenem tissue penetration described previously, we consider that (i) this exposure target in plasma may be underestimated when 40–50% of *f* T > MIC for meropenem is required in infected tissues; and (ii) it is reasonably speculated that a meropenem drug concentration of 1–2 × MIC in plasma for most types of infection but 5 × MIC in plasma for pulmonary, bronchial and pleural infection is sufficient to achieve the pathogen MIC without considering the influence of inflammation on meropenem tissue penetration. Based on all the abovementioned considerations, especially the profiles of meropenem tissue penetration, the targets of 50% *f* T > MIC (mainly for bacterial peritonitis or intraabdominal infections, bloodstream infections, skin and soft tissue infections, or urinary tract infections), 50% *f* T > 2 × MIC (mainly for bacterial hepatitis, metritis, oophoritis, proctitis, or prostatitis, etc.), and 50% *f* T > 5 × MIC (mainly for LRTIs, such as pneumonia, bronchitis, or pleural infections) based on the serum concentration were applied as the optimal PK/PD index in terms of obtaining adequate meropenem exposures regarding its bacterial killing and clinical efficacy for various types of infection or infected sites in the present study.

The %*f* T > MIC in OTAT was calculated using the following one-compartment intravenous infusion equation, as modified from a previously reported equation (Li et al., 2006):

- Meropenem was administered via TSPI;
  $$\begin{array}{l} f\text{T}>\text{MIC}=T_{\inf}-\frac{V_{d}}{CL}\times L\text{n}\left(\frac{R_{0}/CL}{R_{0}/CL-MIC}\right)+\frac{V_{d}}{CL}\times \\ \;L\text{n}\left(\frac{R_{0}/CL-R_{0}/CL\times e^{-CLT_{\inf}/V_{d}}}{MIC}\right) \end{array}$$
  Then
  $$\begin{array}{l} f\text{T}>n\times \text{MIC}=T_{\inf}-\frac{V_{d}}{CL}\times L\text{n}\left(\frac{R_{0}/CL}{R_{0}/CL-n\cdot MIC}\right)+\\ \;\frac{V_{d}}{CL}\times L\text{n}\left(\frac{R_{0}/CL-R_{0}/CL\times e^{-CLT_{\inf}/V_{d}}}{n\cdot MIC}\right) \end{array}$$
- Meropenem was administered via OTAT, and prolonged infusion was started immediately following the completion of IVB (see Figure 1);
  fT>n×MIC=Tinf+VdCL×Ln(f·Dosebol⋅CLVd+f·Doseinf⋅(1−e−CL·Tinf/Vd)/TinfCL⋅n⋅MIC)

and %*f* T > *n* × MIC = *f* T > *n* × MIC × 100/*DI*.

where *f* is the fraction of unbound drug, *f* T is the time that the drug is in unbound (free) form, MIC is the minimum inhibitory concentration, *f* T > MIC is the time that the unbound (free) drug concentrations remain above MIC, *f* T > *n* × MIC is the time that the unbound (free) drug concentrations remain above the MIC by *n*-fold, *n* is an integer set as 1, 2, or 5 in the present study, %*f* T > *n* × MIC is the percentage of the dosing interval during which unbound (free) drug concentrations remain above the MIC by *n*-fold, *T*_inf_ (h) is the infusion time, *R*_0_ (mg/h) is the zero-order infusion rate calculated as whole-dose × *f* /*T*_inf_ in TSPI, *Dose*_bol_ (mg) is the dose administered via IVB in OTAT, *Dose*_inf_ (mg) is the dose administered via prolonged infusion in OTAT, *CL* (L/h) is the plasma clearance rate of the experiment, *V*_d_ (L) is the volume of distribution of the experiment at steady state, *e* is the exponent, *Ln* is the natural logarithm, and *DI* (h) is the dosing interval.

### Meropenem Population PK Parameters

Serum population PK parameters for meropenem were obtained from previously published studies documenting adult patients (preferably those describing infection studies when available) with normal renal function (i.e., creatinine clearance (*CL*_cr_) ≥ 50 ml/min) or healthy volunteers (when the desired data from infected populations were unavailable). Meropenem population PK parameters were determined by the PK model established by Li et al. (2006) as follows: *CL*(L/h) = 14.6 × (*CL*_cr_/83)^0.62^ × (*AGE*/35)^(−0.34)^, *V*_c_(L) = 10.8 × (*WT*/70)^0.99^, and *V*_p_(L) = 12.6 where *CL*_cr_ (ml/min) is the creatinine clearance of the patient calculated according to the Cockcroft-Gault equation based on the patient's ideal body weight (Cockcroft and Gault, 1976), *WT* (kg) is the ideal body weight of the patient, *V*_c_ (L) is the central volume of distribution, and *V*_p_ (L) is the peripheral volume of distribution. The data in this study were chosen for our analysis because compared with other studies (Gonçalves-Pereira and Póvoa, 2011), this study had a relatively large number of patients (*N* = 79), and all of the patients had various types of infections, including 52 patients with intra-abdominal infections, 21 patients with ventilator-associated pneumonia, and six patients with community-acquired pneumonia, and were treated with meropenem; therefore, the PK data obtained for meropenem are relatively representative. The meropenem population PK parameter estimates at steady state based on the demographic characteristics of the subjects in this study are summarized as follows: *CL* 14.97 ± 4.13 L/h and *V*_d_ (*V*_c_ + *V*_p_) 23.86 ± 2.46 L. The ranges of the unbound fraction (*f*) were calculated from the protein binding data, and estimates of *f* for meropenem (0.85–0.98) were obtained from the package insert of the product (USP PACKAGE INSERT., 1996) and from the pharmacokinetic studies (Kuti et al., 2005), if measured.

### Microbiological Susceptibility Data

The microbiological susceptibility data, including the targeted bacterial species, the number of isolates with meropenem MICs ≥ 16 mg/L and the corresponding MIC frequency distributions (in Table 2), were derived from the EUCAST database [European Committee on Antimicrobial Susceptibility Testing (EUCAST), 2019]. This database was chosen for our analysis because it provided the most current and comprehensive collection of MIC data for the antibiotics and organisms modeled in the current study. Bacterial species with isolates having meropenem MICs ≥ 16 mg/L (≥100 strains) include mainly *Acinetobacter baumannii* (763 strains), *Acinetobacter* spp. (1,209 strains), *Enterococcus faecalis* (1,317 strains), *Enterococcus faecium* (1,554 strains), *P. aeruginosa* (4,841 strains), *Staphylococcus epidermidis* (175 strains), *Staphylococcus haemolyticus* (103 strains), and *Stenotrophomonas maltophilia* (3,935 strains). These bacterial species will therefore be modeled as targets for investigating meropenem exposures in monotherapy against MNBSs with MICs ≥ 16 mg/L.

**Table 2:** No. of targeted bacterial isolates and corresponding MIC frequency distributions at MICs ≥16 mg/L collected from the EUCAST database.

**Organism**	**No. of isolates at an MIC of** **≥16 (mg/L**, ***n*****)**							**Corresponding MIC frequency distributions (μg/ml, % of total isolates)**						
	**16**	**32**	**64**	**128**	**256**	**512**	**Total**	**16**	**32**	**64**	**128**	**256**	**512**	**Total**
AB	570	62	59	54	8	10	763	74.71	8.13	7.73	7.08	1.05	1.31	100
AS	1198	10	1	0	0	0	1209	99.09	0.83	0.08	0	0	0	100
EFS	944	168	17	1	0	187	1317	71.68	12.76	1.29	0.08	0	14.20	100
EFM	483	912	5	13	141	0	1554	31.08	58.69	0.32	0.84	9.07	0	100
PA	3959	392	443	33	14	0	4841	81.78	8.10	9.15	0.68	0.29	0	100
SE	152	19	4	0	0	0	175	86.86	10.86	2.29	0	0	0	100
SHA	47	46	9	1	0	0	103	45.63	44.66	8.74	0.97	0	0	100
SM	3820	111	2	2	0	0	3935	97.08	2.82	0.05	0.05	0	0	100

*AB, Acinetobacter baumannii; AS, Acinetobacter spp.; EFS, Enterococcus faecalis; EFM, Enterococcus faecium; PA, Pseudomonas aeruginosa; SE, Staphylococcus epidermidis; SHA, Staphylococcus haemolyticus; SM, Stenotrophomonas maltophilia*.

### Monte Carlo Simulation

A 5,000-subject simulation was performed by Crystal Ball software (version 7.2.2; Decisioneering, Inc., Denver, CO, USA) to calculate the probability of achieving the requisite PK/PD exposure (i.e., 50% *f* T > MIC, 50% *f* T > 2 × MIC, and 50% *f* T > 5 × MIC) for each dosage regimen, referred to as the PTA against the isolates at a specific MIC and the CFR against a population of an organism with a pooled MIC distribution. Prior to the simulations, PK parameters were assumed to follow log-normal distributions and the fraction unbound *f* followed a uniform distribution, whereby the probability was equal within the specified range. The PTA was determined by calculating the fraction of subjects who attained the target at a specific MIC and was determined for MICs between 16 and 512 mg/L. A regimen with a PTA of ≥90% against the isolates at this MIC was considered optimal. The overall expectation value for the PTA (i.e., CFR) is related to PD target attainment in that it expresses the probability of a given dosage regimen achieving the desired exposures against an entire population of pathogens. The CFR percentages for each organism were calculated by multiplying the PTA at each MIC by the percentage of isolates of each of the modeled organisms actually found at that MIC. A regimen with a CFR of ≥90% against an organism population of was considered optimal.

## Results

### Probability of Target Attainment

**Figure 2:**
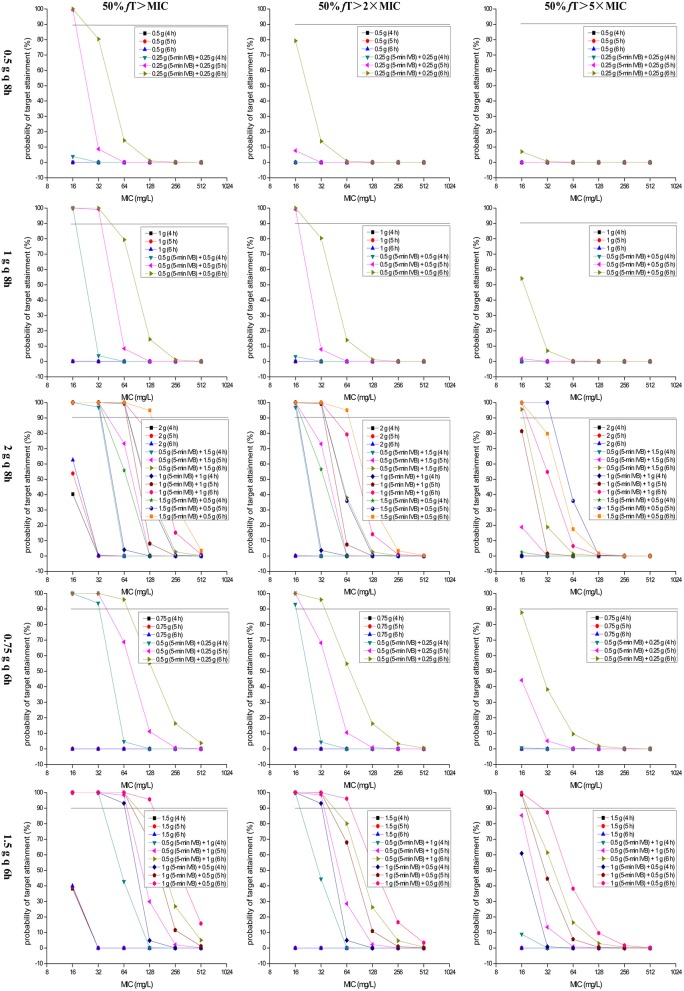
PTAs of achieving 50% *f*T > MIC, 50% *f*T > 2 × MIC and 50% *f*T > 5 × MIC for meropenem with various dosage regimens simulated for MICs up to 512 mg/L. MIC, minimum inhibitory concentration; 50% *f*T > MIC, 50% of the dosing interval during which free drug concentrations remain above the MIC; 50% *f*T > 2 × MIC, 50% of the dosing interval during which free drug concentrations remain above the MIC by two-fold; 50% *f*T > 5 × MIC, 50% of the dosing interval during which free drug concentrations remain above the MIC by five-fold.

PTA vs. MIC profiles for simulations of different dosage regimens are presented in Figure 2. A PTA of ≥90% was considered satisfactory. For attainment of the classical PD target, i.e., 50% *f* T > MIC, the dosage regimens of 0.5 g q 8 h, 1 g q 8 h, 2 g q 8 h, 0.75 g q 6 h, and 1.5 g q 6 h would be insufficient for the treatment of bacteria with MICs ≥ 16 mg/L if administered via 4- to 6-h TSPI, regardless of the MIC values and PD targets. However, they would be adequate if administered as a reasonable OTAT. Specifically, the dosage regimen of 0.5 g [e.g., 0.25 g (5-min IVB) + 0.25 g (5–6 h)] q 8 h for the isolates with MICs of 16 mg/L, 1 g [e.g., 0.5 g (5-min IVB) + 0.5 g (5 h)] q 8 h for the isolates with MICs of 32 mg/L, 0.75 g [e.g., 0.5 g (5-min IVB) + 0.25 g (6 h)] q 6 h for the isolates with MICs of 64 mg/L, 2 g [e.g., 1.5 g (5-min IVB) + 0.5 g (6 h)] q 8 h and 1.5 g [e.g., 1 g (5-min IVB) + 0.5 g (6 h)] q 6 h for the isolates with MICs of 128 mg/L produced sufficient PK/PD exposures when 50% *f* T > MIC was used as the PD target. However, the power of these dosage regimens was weakened by nearly half when 50% *f* T > 2 × MIC was chosen as the PD target and substantially reduced when 50% *f* T > 5 × MIC was chosen as the PD target. Interestingly, the dosage regimen of 2 g [preferred 1.5 g (5-min IVB) + 0.5 g (5 h)] q 8 h still showed good antibacterial properties for the isolates with MICs of up to 32 mg/L even though the highest PD target of 50% *f* T > 5 × MIC was used. Of note, as a modification of the dosage regimen of 2 g q 8 h, the dosage regimen of 1.5 g q 6 h produced a PTA of ≥90% for the isolates with MICs of 16 mg/L when 50% *f* T > 5 × MIC was used as the PD target when it was administered as an OTAT [e.g., 1.5 g [1 g (5-min IVB) + 0.5 g (5 h)] q 6 h], suggesting a potentially useful dosage regimen for these isolates. Table 3 summarizes the coverage of various dosage regimens for the pathogen isolates with meropenem MICs ≥ 16 mg/L in different types of infection at the condition of achieving ≥90% PTA.

**Table 3:** Summary of the coverage of various dosage regimens for the pathogen isolates with MICs of ≥16 mg/L at the condition of achieving ≥90% PTA and/or the targeted pathogen population with pooled MIC distributions between 16 and 512 mg/L at the condition of achieving ≥90% CFR in different types of infection.

**Dosing models**	**Dosage regimen**	**Covered pathogen isolates and/or populations in various types of the infection at different PD targets**		
		**50% *f*T > MIC**	**50% *f*T > 2 × MIC**	**50% *f*T > 5 × MIC**
		**Mainly for bacterial peritonitis or intraabdominal** **infections, bloodstream infections, skin and** **soft tissue infections, or urinary tract infections**	**Mainly for bacterial hepatitis, metritis,** **oophoritis, proctitis, or prostatitis, etc**.	**Mainly for LRTIs, such as** **pneumonia, bronchitis, or** **pleural infections**
0.5 g q 8 h	0.5 g (4 h)	NA	NA	NA
	0.5 g (5 h)	NA	NA	NA
	0.5 g (6 h)	NA	NA	NA
	0.25 g (5-min IVB) + 0.25 g (4 h)	NA	NA	NA
	0.25 g (5-min IVB) + 0.25 g (5 h)	P_16_+(AS, SM)	NA	NA
	0.25 g (5-min IVB) + 0.25 g (6 h)	P_16_+(AS, SE, SM)	NA	NA
1 g q 8 h	1 g (4 h)	NA	NA	NA
	1 g (5 h)	NA	NA	NA
	1 g (6 h)	NA	NA	NA
	0.5 g (5-min IVB) + 0.5 g (4 h)	P_16_+(AS, SM)	NA	NA
	0.5 g (5-min IVB) + 0.5 g (5 h)	P_32_+(AS, PA, SE, SHA, SM)	P_16_+(SE, SM)	NA
	0.5 g (5-min IVB) + 0.5 g (6 h)	P_32_+(AB, AS, EFM, PA, SE, SHA, SM)	P_16_+(AS, SE, SM)	NA
2 g q 8 h	2 g (4 h)	NA	NA	NA
	2 g (5 h)	NA	NA	NA
	2 g (6 h)	NA	NA	NA
	0.5 g (5-min IVB) + 1.5 g (4 h)	P_32_+(AS, SE, SM)	P_16_+(AS, SM)	NA
	0.5 g (5-min IVB) + 1.5 g (5 h)	P_32_+(AS, EFM, PA, SE, SHA, SM)	P_16_+(AS, SE, SM)	NA
	0.5 g (5-min IVB) + 1.5 g (6 h)	P_64_+(AB, AS, EFM, PA, SE, SHA, SM)	P_32_+(AS, PA, SE, SHA, SM)	P_16_+(AS, SM)
	1 g (5-min IVB) + 1 g (4 h)	P_32_+(AS, PA, SE, SHA, SM)	P_16_+(AS, SM)	NA
	1 g (5-min IVB) + 1 g (5 h)	P_64_+(AB, AS, EFM, PA, SE, SHA, SM)	P_32_+(AS, PA, SE, SHA, SM)	NA
	1 g (5-min IVB) + 1 g (6 h)	P_64_+(AB, AS, EFM, PA, SE, SHA, SM)	P_32_+(AS, EFM, PA, SE, SHA, SM)	P_16_+(AS, SE, SM)
	1.5 g (5-min IVB) + 0.5 g (4 h)	P_32_+(AS, PA, SE, SHA, SM)	P_16_+(AS, SE, SM)	NA
	1.5 g (5-min IVB) + 0.5 g (5 h)	P_64_+(AB, AS, EFM, PA, SE, SHA, SM)	P_32_+(AS, PA, SE, SHA, SM)	P_32_+(AS, PA, SE, SHA, SM)
	1.5 g (5-min IVB) + 0.5 g (6 h)	P_128_+(AB, AS, EFM, PA, SE, SHA, SM)	P_64_+(AB, AS, EFM, PA, SE, SHA, SM)	P_16_+(AS, SE, SM)
0.75 g q 6 h	0.75 g (4 h)	NA	NA	NA
	0.75 g (5 h)	NA	NA	NA
	0.75 g (6 h)	NA	NA	NA
	0.5 g (5-min IVB) + 0.25 g (4 h)	P_32_+(AS, SE, SM)	P_16_+(AS, SM)	NA
	0.5 g (5-min IVB) + 0.25 g (5 h)	P_32_+(AS, EFM, PA, SE, SHA, SM)	P_16_+(AS, SE, SM)	NA
	0.5 g (5-min IVB) + 0.25 g (6 h)	P_64_+(AB, AS, EFM, PA, SE, SHA, SM)	P_64_+(AS, PA, SE, SHA, SM)	NA
1.5 g q 6 h	1.5 g (4 h)	NA	NA	NA
	1.5 g (5 h)	NA	NA	NA
	1.5 g (6 h)	NA	NA	NA
	0.5 g (5-min IVB) + 1 g (4 h)	P_32_+(AS, PA, SE, SHA, SM)	P_16_+(AS, SE, SM)	NA
	0.5 g (5-min IVB) + 1 g (5 h)	P_64_+(AB, AS, EFM, PA, SE, SHA, SM)	P_32_+(AS, PA, SE, SHA, SM)	NA
	0.5 g (5-min IVB) + 1 g (6 h)	P_64_+(AB, AS, EFM, PA, SE, SHA, SM)	P_32_+(AB, AS, EFM, PA, SE, SHA, SM)	P_16_+(AS, SE, SM)
	1 g (5-min IVB) + 0.5 g (4 h)	P_64_+(AB, AS, EFM, PA, SE, SHA, SM)	P_32_+(AS, SE, SM)	NA
	1 g (5-min IVB) + 0.5 g (5 h)	P_64_+(AB, AS, EFM, PA, SE, SHA, SM)	P_32_+(AS, EFM, PA, SE, SHA, SM)	P_16_+(AS, SE, SM)
	1 g (5-min IVB) + 0.5 g (6 h)	P_128_+(AB, AS, EFM, PA, SE, SHA, SM)	P_64_+(AB, AS, EFM, PA, SE, SHA, SM)	P_16_+(AS, PA, SE, SM)

*AB, Acinetobacter baumannii; AS, Acinetobacter spp.; EFS, Enterococcus faecalis; EFM, Enterococcus faecium; PA, Pseudomonas aeruginosa; SE, Staphylococcus epidermidis; SHA, Staphylococcus haemolyticus; SM, Stenotrophomonas maltophilia*.

*NA, not applicable*.

*P_16_, P_32_, P_64_, P_128_ signify the pathogen isolates with MICs of 16, 32, 64, and 128 mg/L, respectively*.

*Regimens with P_x_ (x = 16, 32, 64 or 128) + (y) (y = AB, AS, EFM, PA, SE, SHA, SM, or a combination) signify that they are competent for the treatment of infections caused by the pathogen isolates actually found at that MIC if the exact MIC values are available and/or for the treatment of infections caused by the targeted pathogen population identified with only bacterial species if the exact MIC values are unavailable*.

### Cumulative Fraction of Response

**Figure 3:**
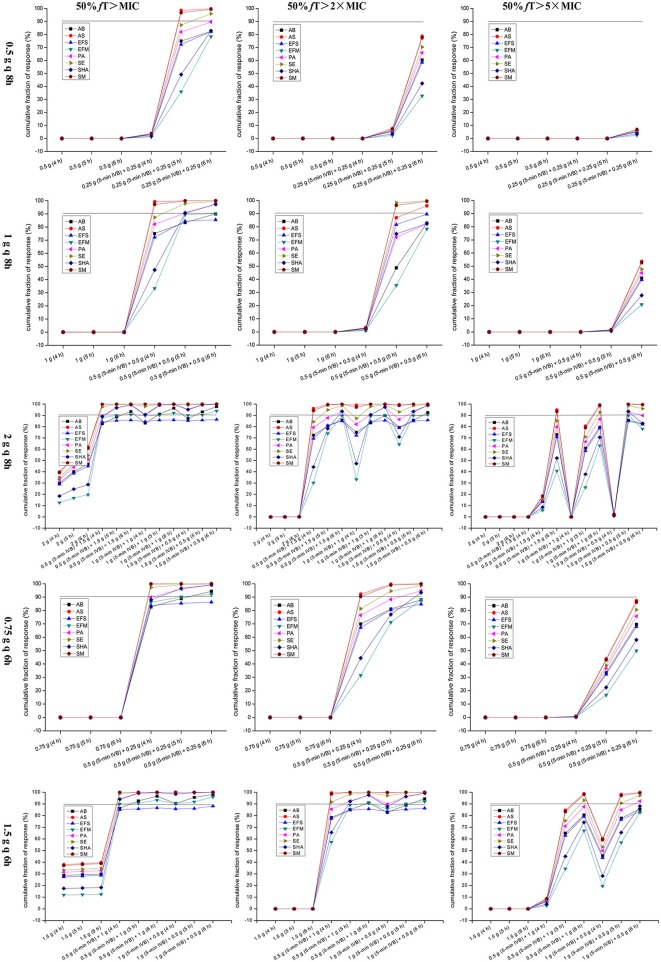
CFRs of achieving 50% *f*T > MIC, 50% *f*T > 2×MIC, and 50% *f*T > 5 × MIC for meropenem with various dosage regimens simulated for the targeted bacteria populations with pooled MIC distributions between 16 and 512 mg/L. AB, *Acinetobacter baumannii*; AS, *Acinetobacter* spp.; EFS, *Enterococcus faecalis*; EFM, *Enterococcus faecium*; PA, *Pseudomonas aeruginosa*; SE, *Staphylococcus epidermidis*; SHA, *Staphylococcus haemolyticus*; SM, *Stenotrophomonas maltophilia*; 50% *f*T > MIC, 50% of the dosing interval during which free drug concentrations remain above the MIC; 50% *f*T > 2 × MIC, 50% of the dosing interval during which free drug concentrations remain above the MIC by two-fold; 50% *f*T > 5 × MIC, 50% of the dosing interval during which free drug concentrations remain above the MIC by five-fold.

CFR vs. various targeted pathogen populations for simulations of different dosage regimens are displayed in Figure 3. A CFR of ≥90% was considered optimal. Obviously, only regimens with OTAT achieved a CFR of ≥90% for the targeted pathogen population, regardless of the PD targets and dosing models. Based on currently pooled MIC distributions, when 50% *f* T > MIC was chosen as the PD target, the dosage regimen of 0.5 g [preferred 0.25 g (5-min IVB) + 0.25 g (6 h)] q 8 h yielded a CFR of ≥90% for only the *Acinetobacter* spp., *S. epidermidis*, and *S. maltophilia* populations; however, the dosage regimens of 1 g [preferred 0.5 g (5-min IVB) + 0.5 g (6 h)] q 8 h, 2 g [e.g., 0.5 g (5-min IVB) + 1.5 g (6 h)] q 8 h, 0.75 g [preferred 0.5 g (5-min IVB) + 0.25 g (6 h)] q 6 h, and 1.5 g [e.g., 0.5 g (5-min IVB) + 1 g (5 h)] q 6 h for all of the targeted pathogen populations achieved the requisite CFR, with *E. faecalis* being the sole exception. When using a higher PD target of 50% *f* T > 2 × MIC, the majority of the simulated dosage regimens had decreased coverage of the targeted pathogens population at the condition of achieving ≥90% CFR. However, the dosage regimens of 2 g [preferred 1.5 g (5-min IVB) + 0.5 g (6 h)] q 8 h and 1.5 g [preferred 0.5 g (5-min IVB) + 1 g (6 h) or 1 g (5-min IVB) + 0.5 g (6 h)] q 6 h still covered the *A. baumannii, Acinetobacter* spp., *E. faecium, P. aeruginosa, S. epidermidis, S. haemolyticus*, and *S. maltophilia* populations at this condition. However, when using an aggressive PD target of 50% *f* T > 5 × MIC, these simulated dosage regimens had a drastically decreased coverages of the targeted pathogen populations at the condition of achieving ≥90% CFR. Surprisingly, however, the dosage regimen of 2 g [1.5 g (5-min IVB) + 0.5 g (5 h)] q 8 h still reached ≥90% CFR for the *Acinetobacter* spp., *P. aeruginosa, S. epidermidis, S. haemolyticus*, and *S. maltophilia* populations. Table 3 summarizes the coverage of various dosage regimens for the targeted bacterial population with pooled MIC distributions between 16 and 512 mg/L in different types of infection at the condition of achieving ≥90% CFR.

## Discussion

To the best of our knowledge, this study is the first to analyze meropenem as a monotherapy aimed at MNBSs with MICs ≥ 16 mg/L. In this study, we considered the tissue penetration profiles of meropenem for infections at different sites when establishing the PK/PD model associated with clinical response. We believe that this study is worthwhile because the PK/PD outcomes generated from our data could help clinicians treat these isolates more effectively through optimization of dosage regimens, especially when better treatment options are absent. Notably, our results support that meropenem as a monotherapy is still competent for isolates with MICs ≥ 16 mg/L provided that the drug is administered as a reasonable OTAT but not as the currently widely recommended TSPI.

### TSPI vs. OTAT for Meropenem Against Highly Resistant Bacterial Isolates

Currently, the optimal meropenem dosage is undergoing ardent evaluation to develop new strategies to overcome increasing meropenem resistance and maximally preserve the effectiveness of this drug. TSPI for meropenem has often been the preferred optimal mode. Indeed, TSPI for meropenem against central nervous system infections due to *P. aeruginosa* (Capitano et al., 2004) or *S. marcescens* (Nicasio et al., 2007) and ventilator-associated pneumonia due to gram-negative bacilli and for treating neutropenic patients with fever have resulted in successful clinical responses when compared with those resulting from intermittent infusion (Lorente et al., 2006; Fehér et al., 2014). However, the benefits of TSPI for meropenem reported by these studies were found using meropenem-susceptible bacterial strains. However, for infections due to MNBSs, especially those with MICs ≥ 16 mg/L, the benefit of this approach for improving clinical efficacy is unknown, and clinical data are limited.

Indeed, this approach is unfavorable for highly resistant bacterial isolates because it reduces the initial bactericidal effects due to both the decrease in meropenem peak concentration and the delay in its peak time, as demonstrated by Eguchi et al. (2010). It is therefore difficult for meropenem to achieve the MIC of highly resistant bacterial isolates, even at a high dose. This reduction in initial bactericidal effects may also be the reason why some studies found that meropenem, even at 3 g/day (e.g., 1 g q 8 h over a 3-h infusion or 0.5 g every 4 h (q 4 h) over a 4-h infusion), displayed poor PK/PD exposures for isolates with MICs ≥ 16 mg/L but showed a relatively good effect for isolates with MICs ≤ 8 mg/L (Vourli et al., 2016; Zhao et al., 2017), thus inferring that TSPI for meropenem may be beneficial for isolates with low MICs. Likewise, our data confirmed this inference because using TSPI for meropenem, even at a high dose of 2 g q 8 h and using 50% *f* T>MIC as the PD target, did not yield a PTA ≥ 90% for isolates with MICs of 16 mg/L. Thus, this conventional approach of using TSPI to optimize PD exposure is rendered futile with the emergence of higher MICs (Avery and Nicolau, 2018). For the data obtained herein, OTAT is preferable because it provides a higher meropenem exposure relative to that of TSPI regardless of the MIC values, dosing models and PD targets. Additionally, meropenem administered via a loading dose of 0.5 g over a 30-min infusion followed immediately by 0.5 g q 4 h over a 4-h infusion [i.e., an OTAT regimen of 0.5 g (0.5 h) + 0.5 g (4 h)] reportedly achieved better outcomes than that administered intermittently against bacteria of intermediate susceptibility (Zhao et al., 2017), thus demonstrating the superiority of OTAT for meropenem against MNBSs.

### Competence of Meropenem in Monotherapy Against Highly Resistant Bacterial Isolates

Currently, clinical experience with meropenem monotherapy for MNBSs with MICs ≥ 16 mg/L is indeed limited because from a clinical point of view, determination of meropenem MICs ≥ 8 mg/L for the identified stains by susceptibility tests causes the vast majority of clinicians to switch to other better options. However, previous studies indicated that meropenem using a high dose and TSPI (e.g., 2 g q 8 h over a 2- to 3-h infusion) in monotherapy can provide some therapeutic benefit and therefore be considered to treat infections due to MNBSs with MICs ≤ 4 or even ≤ 8 mg/L based on the therapeutic efficacy of *K. pneumoniae* carbapenemase (KPC)-producing *K. pneumoniae* (Daikos and Markogiannakis, 2011; Tzouvelekis et al., 2012; Hsu and Tamma, 2014; Tumbarello et al., 2015). Inconsistent with these findings, on a theoretical basis, our data indicated that even for the isolated *K. pneumoniae* strains with meropenem MICs of up to 32 mg/L and utilization of an aggressive PD target of 50% *f* T > 5 × MIC, meropenem in monotherapy, even at the same daily dose used in the abovementioned studies, can still produce desired PK/PD exposures provided that it is administered using the dosage regimen of 2 g [1.5 g (5-min IVB) + 0.5 g (5 h)] q 8 h, as shown in Figure 2.

Regarding the treatment of infections due to MNBSs with MICs ≥ 16 mg/L, most reports currently focus on MCCT for meropenem-nonsusceptible *K. pneumoniae*. Joint guidelines prepared by the Working Party of the British Society for Antimicrobial Chemotherapy, the Healthcare Infection Society and the British Infection Association (Hawkey et al., 2018) considered that MCCT including a high dose and continuous infusion of meropenem would be appropriate for *K. pneumoniae* with MICs > 8 and <64 mg/L. In addition, some studies have indicated that MCCT may grant a survival benefit relative to that of meropenem in monotherapy when the MIC of *K. pneumoniae* is <16 mg/L (Tumbarello et al., 2012; Daikos et al., 2014). Moreover, even for strains with MICs ≥ 16 mg/L, MCCT including a high dose of 13.2 g/day and continuous infusion of meropenem with levels optimized by therapeutic drug monitoring were helpful in obtaining a favorable clinical outcome for infections due to KPC-producing *K. pneumoniae* with MICs of 16–64 mg/L (Pea et al., 2017). Another study also demonstrated the benefits of MCCT using a high dose of 6 g daily and 3-h extended infusion for meropenem for an infection due to meropenem-nonsusceptible *K. pneumoniae* strains with MICs ≥16 mg/L (Giannella et al., 2018).

Understandably, these reports imply that MCCT is preferable for treating meropenem-nonsusceptible *K. pneumoniae* infections arising from strains with MICs > 8 or even ≥16 mg/L. Likewise, some retrospective, prospective observational cohort and multicentric studies have confirmed the superiority of MCCT over monotherapy for the treatment of infections due to KPC-producing *K. pneumoniae*, especially for bloodstream infections (Zarkotou et al., 2011; Qureshi et al., 2012; Tumbarello et al., 2012; Daikos et al., 2014). In contrast, another most recently retrospective study (Kuti et al., 2019), in which PD exposures of meropenem as an adjunctive treatment in plazomicin-based combination therapy (i.e., plazomicin plus meropenem) were evaluated to investigate its synergy in combination against meropenem-nonsusceptible *K. pneumoniae* with MICs ≥ 64 mg/L, showed PD exposures of 0% for meropenem at the target of ≥40% *f* T > MIC when meropenem was administered as 2 g q 8 h over a 3-h infusion and therefore concluded that plazomicin monotherapy was sufficient and optimization of meropenem therapy was not required for the combination to achieve microbiological response and clinical efficacy against serious meropenem-nonsusceptible *K. pneumoniae* infections, including bloodstream infections, hospital acquired pneumonia or ventilator-associated pneumonia.

It should also be noted that because most conclusions on the superiority of MCCT reached from the abovementioned reports were derived based on meropenem-nonsusceptible *K. pneumoniae*, the extrapolation of MCCT using a high dose and prolonged infusion for meropenem to other MNBSs with different resistance mechanisms requires further investigation. Coincidentally, the recent Amsterdam Investigator-Initiated Absorb Strategy All-Comers trial (Paul et al., 2018), in which a randomized, controlled, superiority trial was conducted at six hospitals to investigate the superiority of MCCT (i.e., colistin plus meropenem) vs. colistin alone, showed no differences in the outcomes of patients treated with MCCT or colistin monotherapy for infections (including bloodstream infections, ventilator-associated pneumonia and/or hospital acquired pneumonia, and urinary tract infections) due to carbapenem-resistant *Acinetobacter* spp., *Enterobacteriaceae*, and *P. aeruginosa* in which 97% of the isolates had meropenem MICs > 8 mg/L. This result supported that colistin monotherapy is equipotent to MCCT for such infections, and it is therefore unnecessary to use MCCT to treat infections due to these MNBSs. Overall, the choice of MCCT or monotherapy for the treatment of infections due to MNBSs remains a matter of debate. Also, it is important to note that co-administration of different antibiotics may lead to important concomitant adverse effects, including *Clostridium difficile* infection, selection of further resistances, or nephrotoxicity (Petrosillo et al., 2013).

Inconsistent with these reports, our data supported that even meropenem monotherapy, rather than plazomicin monotherapy, colistin monotherapy, or MCCT, is sufficient for the treatment of such infections due to these pathogens (i.e., the abovementioned *Acinetobacter* spp., *P. aeruginosa*, and *Enterobacteriaceae*, including meropenem-nonsusceptible *K. pneumoniae*) and for situations in which meropenem exposures are not reduced relative to plasma exposures, such as bloodstream infections, skin and soft tissue infections, and urinary tract infections. Meropenem monotherapy, in which it must be administered at a high dose of 2 g q 8 h or 1.5 g q 6 h and as an OTAT, can achieve optimal PK/PD exposures for MNBSs with MICs of up to 128 mg/L and for pathogen populations with a pooled MIC distribution, as demonstrated by achieving a PTA of ≥90% at an MIC of 128 mg/L and a CFR of ≥90% for *Acinetobacter* spp., *P. aeruginosa*, and *Enterobacteriaceae* populations when using the dosage regimen of 2 g [1.5 g (5-min IVB) + 0.5 g (6 h)] q 8 h or 1.5 g [1 g (5-min IVB) + 0.5 g (6 h)] q 6 h and using 50% *f* T > MIC as the PD target for such types of infection.

However, meropenem monotherapy did not display acceptable PK/PD exposures for the isolates with MICs ≥ 256 mg/L in the present study regardless of the dosing models and PD targets. Interestingly, another PK/PD study (Del Bono et al., 2017) in which meropenem using a high dose of 2 g q 8 h and 3-h TSPI in MCCT (i.e., meropenem plus tigecycline or gentamicin or colistin, or meropenem plus tigecycline plus gentamicin or colistin) was used to treat bloodstream infections due to KPC-producing *K. pneumoniae* with actual meropenem MICs ≥ 256 mg/L also showed that meropenem did not achieve the PD target of *T* > 40% 1 × MIC in these isolates based on the measured meropenem levels despite the MCCT used. Moreover, at this dosage condition, meropenem could have attained PTAs of only 68 and 32% at that PD target in isolates with hypothetical MICs of 16 and 32 mg/L, respectively, despite the MCCT used. Thus, no synergisms were concluded between meropenem and the co-administered agents in this study. This conclusion was confirmed by the study conducted by Kuti et al. (2019), in which meropenem administered as 2 g q 8 h over a 3-h infusion had no synergy on the co-administered plazomicin when used for the treatment of infections due to meropenem-nonsusceptible *K. pneumoniae* with meropenem MICs ≥64 mg/L. One reason why unsatisfactory meropenem exposures were observed in these studies may be that TSPI for meropenem reduced its AUC exposures above the MIC, especially for values >16 mg/L, due to both the decrease in its peak concentration and the delay in its peak time. Based on these observations and ours, when administered as a reasonable OTAT and used for bloodstream infections, meropenem monotherapy displayed equally discontented exposures against highly resistant bacterial isolates with MICs ≥ 256 mg/L but showed a superior effect against relatively lowly resistant bacterial isolates with MICs ≤ 128 mg/L when compared with that of MCCT including a high dose and TSPI of meropenem. This finding suggests a possible usefulness of meropenem monotherapy for the treatment of such infections caused by isolates with MICs of up to 128 mg/L.

High MICs often result in insufficient antimicrobial tissue exposures, especially when the drug is used in inappropriate dosage regimens and used for infected sites with poor drug penetration. Regarding this issue, our data provide very useful directions on whether meropenem at the optimal dosage regimens can be used as a monotherapy for the treatment of infections due to isolates with high MICs at different sites based on the exact MICs if available. Given the profiles of meropenem tissue penetration, for infections due to isolates with MICs ≥ 16 mg/L occurring in the liver, skin, uterus, ovaries, rectum, prostate, trachea, etc., meropenem monotherapy with at least a 3 g daily dose yields good outcomes, e.g., the dosage regimen of 1 g [preferred 0.5 g (5-min IVB) + 0.5 g (5–6 h)] q 8 h for isolates with MICs of 16 mg/L, the dosage regimen of 2 g [preferred 1.5 g (5-min IVB) + 0.5 g (6 h)] q 8 h, 0.75 g [preferred 0.5 g (5-min IVB) + 0.25 g (6 h)] q 6 h or 1.5 g [preferred 1 g (5-min IVB) + 0.5 g (6 h)] q 6 h for isolates with MICs of up to 64 mg/L, and is thus sufficient. Satisfactorily, for infections occurring in the peritoneum, the dosage regimen of 0.5 g [preferred 0.25 g (5-min IVB) + 0.25 g (5–6 h)] q 8 h would be sufficient for isolates with MICs of 16 mg/L, and the dosage regimen of 1 g [preferred 0.5 g (5-min IVB) + 0.5 g (5–6 h)] q 8 h, 0.75 g [preferred 0.5 g (5-min IVB) + 0.25 g (6 h)] q 6 h, 2 g [preferred 1.5 g (5-min IVB) + 0.5 g (6 h)] q 8 h or 1.5 g [preferred 1 g (5-min IVB) + 0.5 g (6 h)] q 6 h for isolates with MICs of up to 32, 64, and 128 mg/L would be adequate, respectively. Surprisingly, even for infections located in the lung, bronchus, and pleura, meropenem with the preferred regimen of 2 g [1.5 g (5-min IVB) + 0.5 g (5 h)] q 8 h still proved effective for isolates with MICs of up to 32 mg/L, suggesting that for LRTIs and pleural infections, meropenem monotherapy can still perform good bactericidal action on isolates with MICs of up to 32 mg/L.

However, the exact MIC values, especially those >16 mg/L, are often unavailable because automated systems such as VITEK-2, which indicate MIC values as high as >16 mg/L, are unable to determine the precise MIC. Regarding this problem, our data provided the CFR for the targeted bacterial population and summarized the treatment options (Table 3). As a potential monotherapy, meropenem with an aggressive dosage regimen of 2 g [preferred 1.5 g (5-min IVB) + 0.5 g (6 h)] q 8 h or 1.5 g [preferred 1 g (5-min IVB) + 0.5 g (6 h)] q 6 h may be the best choice and is worth trying in its empiric therapy, especially for critically ill patients, because even at a PD target of 50% *f* T > 2 × MIC, these dosage regimens could produce a CFR of ≥90% for all of the tested bacterial populations with MICs ≥16 mg/L, including *A. baumannii, Acinetobacter* spp., *E. faecium, P. aeruginosa, S. epidermidis, S. haemolyticus*, and *S. maltophilia*, with the sole exception of *E. faecalis*. This result suggests that in the empiric therapy of meropenem, these dosage regimens would be competent for the vast majority of infections (e.g., bloodstream infections, intraabdominal infections, skin, and soft tissue infections, and urinary tract infections) due to these pathogens based on the profiles of meropenem tissue penetration. However, for LRTIs and pleural infections, meropenem monotherapy with the most promising dosage regimen of 2 g [1.5 g (5-min IVB) + 0.5 g (5 h)] q 8 h would be sufficient for infections due to only *Acinetobacter* spp., *P. aeruginosa, S. epidermidis, S. haemolyticus*, and *S. maltophilia*. Table 4 summarizes some preferred dosage regimens in the empiric therapy of meropenem for different types of infection based on our analysis.

**Table 4:** Summary of preferred treatment option recommendations in the empiric therapy of meropenem for different types of infection based on our analysis.

**Optimal PD target**	**Corresponding types of infection or infected sites**	**Preferred treatment option recommendations for MNBSs with MICs ≥16 mg/L**
50% *f*T > MIC	Bacterial peritonitis or intraabdominal infections, bloodstream infections, skin and soft tissue infections, or urinary tract infections	2 g [1.5 g (5-min IVB) + 0.5 g (6 h)] q 8 h or 1.5 g [(5-min IVB) + 0.5 g (6 h)] q 6 h
50% *f*T > 2 × MIC	Bacterial hepatitis, metritis, oophoritis, proctitis, or prostatitis	2 g [1.5 g (5-min IVB) + 0.5 g (6 h)] q 8 h or 1.5 g [1 g (5-min IVB) + 0.5 g (6 h)] q 6 h
50% *f*T > 5 × MIC	LRTIs, such as pneumonia, bronchitis, or pleural infections	2 g [1.5 g (5-min IVB) + 0.5 g (5 h)] q 8 h

Of note, these optimal dosage regimens sufficient for MNBSs are also adequate for meropenem-susceptible bacterial strains since the regimens established at higher MICs generate higher PTAs and CFRs at low MICs. In addition, we need to monitor renal function regularly and adjust the meropenem dose as required, particularly for the high dose regimens, as the majority of the patients infected with meropenem-nonsusceptible *K. pneumoniae* are critically ill and have altered renal function (Daikos and Markogiannakis, 2011). Although meropenem monotherapy displays satisfactory PK/PD exposures against highly resistant bacterial isolates, especially when it is administered at a high dose of 2 g q 8 h in OTAT, the accompanying safety issues are also worthy of our attention, especially when a high dose is used. However, although the most frequent adverse events associated with meropenem use, such as diarrhea, rash, nausea, and vomiting, thrombocytosis, eosinophilia and changes in hepatic biochemistry and the possible episodes of seizures are reported, meropenem exhibits an acceptable safety profile with good central nervous system and gastrointestinal tolerability, even at a high dose of up to 6 g per day (2 g q 8 h), and shows also a favorable safety profile in a number of special patient populations, including elderly, renally impaired, pediatric, and neutropenic patients, patients with cystic fibrosis and those with meningitis (Norrby, 1995; Norrby et al., 1995; Norrby and Gildon, 1999; Linden, 2007). Therefore, the dosage regimens recommended herein for highly resistant bacterial strains should be safe and worthwhile to try.

### Study Limitations

The present study has some limitations. First, our data did not include meropenem concentrations in plasma or infected sites. Second, the results of simulation analysis were not validated by evaluating clinical outcomes, limiting the generalization of the conclusions. Third, the susceptibility data used in the modeling for our predictions of target attainment were obtained from the EUCAST database. As such, our findings should be interpreted and extrapolated while paying attention to the different or changing susceptibility profiles in one's own hospital. Fourth, a one-compartment model was used to calculate the meropenem PD exposures, whereas other studies have suggested that its *in vivo* pharmacokinetic disposition is best fitted using a two-compartment model. However, studies on its PK have been published using both one- and two-compartment models as well as non-compartmental analysis (Christensson et al., 1992; Leroy et al., 1992a,b).

Despite these limitations, our data are believable and instructive for prescribers because it considered the profiles of meropenem tissue penetration, used the more representative PK parameters, and integrated the most current and comprehensive MIC data. Importantly, even with *f* (C_min_)/MIC > 5 recommended by Li et al. (2007) as a PD target associated with clinical response, our data derived from 50% *f* T > 5 × MIC also confirmed that meropenem monotherapy is competent for infections due to isolates with MICs of up to 32 mg/L, and importantly in the absence of alternative treatments, our data provide very useful directions on how to choose an optimal dosage regimen for meropenem monotherapy for the treatment of infections due to isolates with high MICs at different sites. To the best of our knowledge, this is the first PD analysis in which meropenem was used as a monotherapy aimed at MNBSs with MICs ≥ 16 mg/L and in which the profiles of meropenem tissue penetration were taken into account for setting the PD targets and calculating the PTAs and CFRs. In addition, we plan to perform a clinical trial to confirm our findings and to validate the optimal dosage by using the established equation for %*f* T > MIC and the Monte Carlo simulation.

## Conclusions

When faced with the daily challenge of infections due to MNBSs, we should try to reduce the gap between the available medical evidence for using meropenem against such infections and the dearth of alternative therapeutic options, some of which have not been sufficiently explored and/or whose efficacy in certain situations remains doubtful. Whether we can continue to use meropenem in the presence of MNBSs with high MICs remains controversial. The data analyses presented herein support the opinion that meropenem monotherapy can still be considered for use against MNBSs provided that (i) the MIC for the infecting pathogen isolates is ≤ 32 mg/L and (ii) a reasonable OTAT is used to drive the PK/PD profiles to acceptable exposures. However, in the absence of control trials, the continued appraisal of meropenem for use as a monotherapy, along with the optimal dosage regimens in clinical experience, will provide further important information on its utility against MNBSs.

## Data Availability Statement

All datasets generated for this study are included in the manuscript.

## Author Contributions

XS performed the model simulation and wrote the manuscript. YW, LC, DY, and ML conceptualized and supervised the manuscript. All authors approved the final version of the manuscript.

### Conflict of Interest

The authors declare that the research was conducted in the absence of any commercial or financial relationships that could be construed as a potential conflict of interest.

## Abbreviations

MNBSs: meropenem-nonsusceptible bacterial strains
MIC: minimum inhibitory concentration
MCCT: meropenem-containing combination therapy
TSPI: traditional simple prolonged-infusion
OTAT: optimized two-step-administration therapy
PK: pharmacokinetic
PD: pharmacodynamic
EUCAST: European Committee on Antimicrobial Susceptibility Testing
PTAs: probabilities of target attainment
CFRs: cumulative fractions of response
IVB: i.v. bolus
AUC: area under the concentration-time curve
ELF: epithelial lining fluid
*f* T > MIC: the time that the unbound (free) drug concentrations remain above the minimum inhibitory concentration
%*f* T > MIC: percentages of the dosing interval during which unbound (free) drug concentrations remain above the MIC
LRTIs: lower respiratory tract infections
*f* (C_min_)/MIC: ratio of the trough concentration of unbound (free) drug to the minimum inhibitory concentration
*CL*_cr_: creatinine clearance
KPC: *Klebsiella pneumoniae* carbapenemase.
